# A Novel Mutation in *DDR2* Associated with Warburg-Cinotti Syndrome in a Neonate

**DOI:** 10.34172/aim.34458

**Published:** 2025-10-01

**Authors:** Junping Xiao, Chenyu Zhuan, Lingkong Zeng, Xuwei Tao

**Affiliations:** ^1^Department of Neonatology, Wuhan Women and Children Medical Care Center, Tongji Medical College, Huazhong University of Science and Technology, Wuhan, China

**Keywords:** Discoidin domain, Discoidin domain receptor 2, Neonatal respiratory distress, Warburg-Cinotti syndrome

## Abstract

Warburg-Cinotti syndrome (WCS) is a rare disorder caused by mutations in the DDR2 gene. We report the first neonatal case with a novel WCS variant, aiming to explore its clinical and genetic characteristics. Clinical data were collected and analyzed retrospectively, and whole exome sequencing (WES) was performed for the family. The patient exhibited significant respiratory distress due to choanal abnormalities, unlike previous reports. WES revealed a maternally inherited heterozygous missense mutation in *DDR2* (c.431A>G, p.Asn144Ser). *In-vitro* experiments showed that the mutated *DDR2* fails to activate the p38 MAPK pathway. The study suggests that this novel mutation may contribute to the patient’s condition, especially in the neonatal period, and may expand the phenotypic spectrum, providing new references for clinical diagnosis and gene therapy.

## Introduction

 Warburg-Cinotti syndrome (WCS, MIM: 618175) is a rare inherited autosomal dominant disorder caused by mutations in the discoidin domain receptor 2 (*DDR2*) gene. The first reported case involved a male patient presenting with a range of symptoms, including corneal pannus, conductive hearing loss, hand joint flexion conjectures, phalangeal osteolytic defects, joint lesions, significant fat loss in the hands, feet, and face, oligospermia, spontaneous pneumothorax, and facial dysmorphism.^1^ This case was pivotal in recognizing WCS as a novel syndrome. Subsequently, another male patient with similar clinical features was reported.^2^ A comprehensive review analyzed six cases further expanded the phenotype spectrum of WCS, and confirmed its significant association with specific *DDR2* missense- p.Leu610Pro and p.Tyr740Cys. These mutations, located in highly conserved regions of the *DDR2* amino acid sequence, were validated through genetic functional assessments and have been implicated in various physiological and pathological conditions, including impaired ovulation, reduced sperm production, aberrant bone formation, abnormal angiogenesis, organ fibrosis, osteoarthritis, epithelial tissue tumors, and delayed wound healing.^3^

 Our case represents the first reported instance of WCS presenting in the neonatal period, with genetic functional testing revealing a *DDR2* mutation potentially associated with the condition. Our findings expand the known clinical phenotype of WCS and offer valuable insights for early diagnosis and gene therapy in neonates with this syndrome.

## Case Report

 The patient was a 2-day-old term male infant who presented with respiratory distress immediately after birth. The infant was delivered vaginally (G2P1) with Apgar scores of 9, 10, and 10 at 1, 5, and 10 minutes, respectively. His birth weight was 2.8 kg (10^th^ percentile, -8.4 SD), head circumference was 36 cm (97^th^ percentile, + 1.7 SD), and length was 53 cm (97^th^ percentile, + 1.6 SD). The amniotic fluid, umbilical cord, and placenta were all normal. Despite initial intubation and mechanical ventilation in the local neonatal intensive care unit, the infant showed no signs of improvement after two days and was subsequently transferred to our ward.

**Figure 1:**
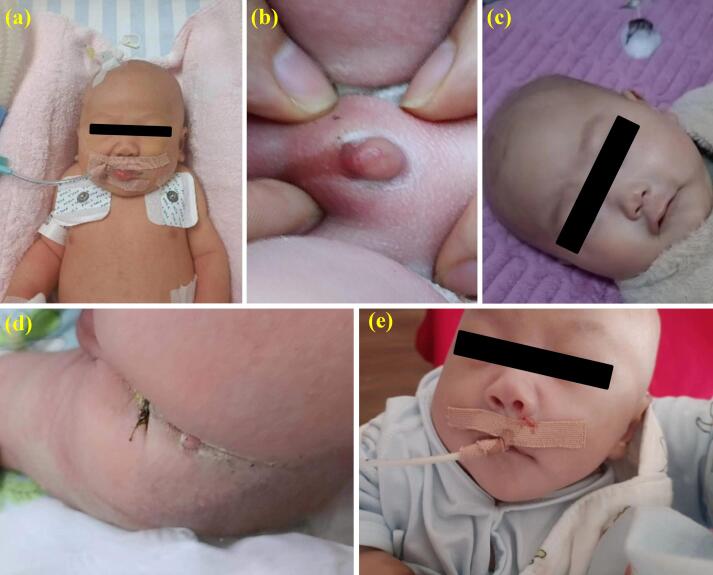
Physical Characteristics of the Case. (a) Short neck with webbing; (b) Microphallus; (c) Low nasal bridge; (d) Mass at the sacral region; (e) Lower-set ears

 Upon admission, the patient’s physical examination revealed a temperature of 36.8 °C, heart rate of 146 beats/min, and blood pressure of 62/46 mm Hg. Notable findings included a short chin, a short neck with evident webbing, and lower-set ears. The thoracic cavity appeared well-developed, but decreased breath sounds were noted in the left lung compared to the right. A grade-I continuous systolic murmur was detected in the precordial area. Muscle tone in the limbs was elevated, with restricted voluntary movements. The penis measured approximately 5 mm (-4.25 SD) in length. A soft, solid mass measuring 6 × 5 mm was palpated in the sacral region, with overlying skin showing no sinus tracts, hair coverage, discharge, or signs of erythema, swelling, heat, or pain. These abnormalities are illustrated in Figure 1.

**Figure 2:**
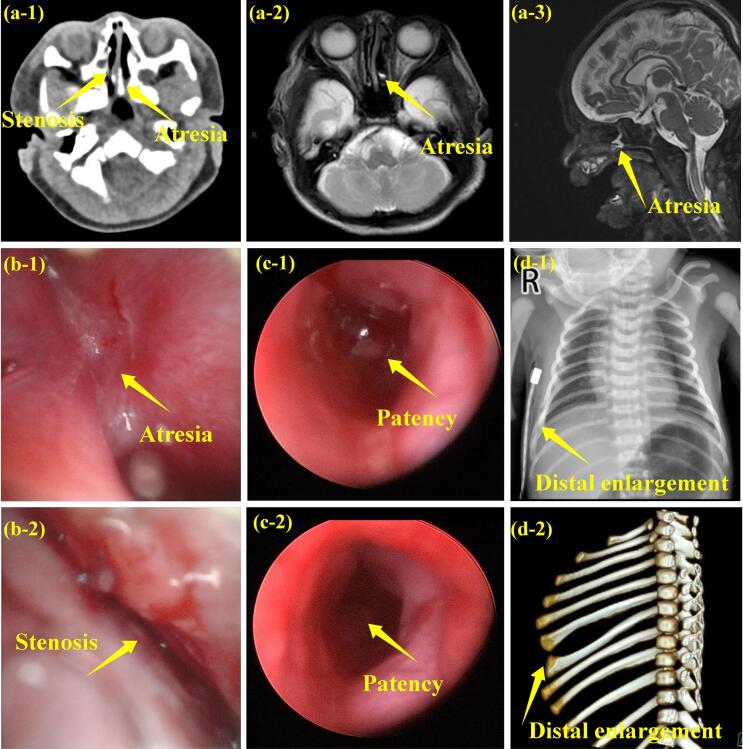
Imaging Findings of the Case.(a) Nasal abnormalities by radiographic examination: left choanal atresia and right posterior nasal stenosis (a-1: CT-scan, a 2-3: MRI);(b) Nasal abnormalities by fiberoptic bronchoscopy (b1-2: after birth, b3-4: after operation);(c) Distal enlargement of the right 6th and 7th ribs (c-1: X-ray, c-2: CT-scan)

 Laboratory investigations, including complete blood count, biochemical parameters, blood amino acids, urine organic acids, and cultures of blood and sputum, revealed no significant abnormalities. Ultrasound imaging indicated the presence of an atrial septal defect measuring approximately 2.4 mm, potential hypoplastic testicles, and laxity in the left hip joint. CT scans revealed nasal malformations, specifically left choanal atresia and right posterior nasal stenosis, which were further confirmed by fiberoptic bronchoscopy and cranial MRI. Additionally, distal enlargement of the right 6th and 7th ribs was noted on CT imaging (Figure 2).

**Figure 3:**
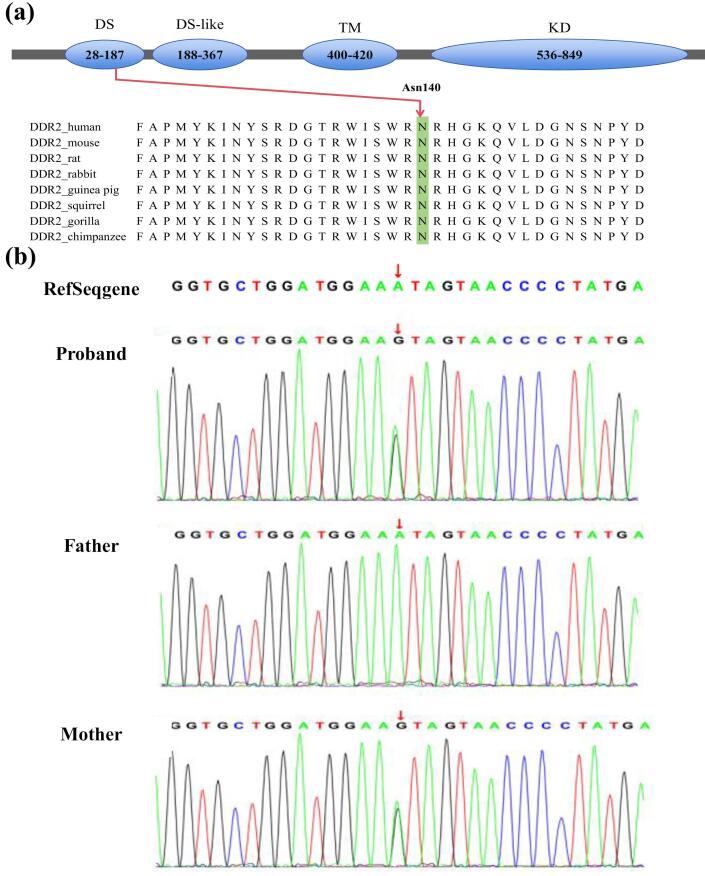
(a) Domains structure diagram of *DDR2; *(b) Whole exome sequencing of the family

 Whole exome sequencing (WES) identified a maternally inherited heterozygous missense genomic variant, NM_006182.4(*DDR2*):c.431A > G, resulting in an amino acid substitution p.(Asn144Ser) (Figure 3). The affected asparagine residue at position 144 is located within the N-terminal of *DDR2*, which is highly conserved across vertebrate and invertebrate homologs. This case involves a maternally inherited *DDR2* mutation. The mother has congenital polydactyly but presents with no other abnormalities. The mother exhibited mild or no symptoms (incomplete penetrance), suggesting that maternal modifiers and environmental factors may significantly mitigate the phenotypic severity of this mutation.

 Upon admission, the patient was initiated on mechanical ventilation and received comprehensive supportive care, including anti-infective therapy and enteral nutrition. (Video S1 shows the respiratory distress; see Supplementary file 1). Following the diagnosis of choanal deformities the patient underwent surgical intervention on the 10^th^ day of hospitalization (Figure 2) and was discharged after 25 days of treatment. During the 9-month follow-up, the patient developed milia-sized hypopigmented macules on both hands, accompanied by erythema, papules, and scratch marks on both knee joints, as well as rib flaring. Developmental delays were noted, with measurements indicating a weight of 8100 g (5^th^-10^th^ percentile), head circumference of 41.9 cm (less than 3^rd^ percentile), and height of 67.5 cm (3^rd^ percentile). Unfortunately, the parents declined to provide any additional information regarding the patient’s condition.

**Figure 4:**
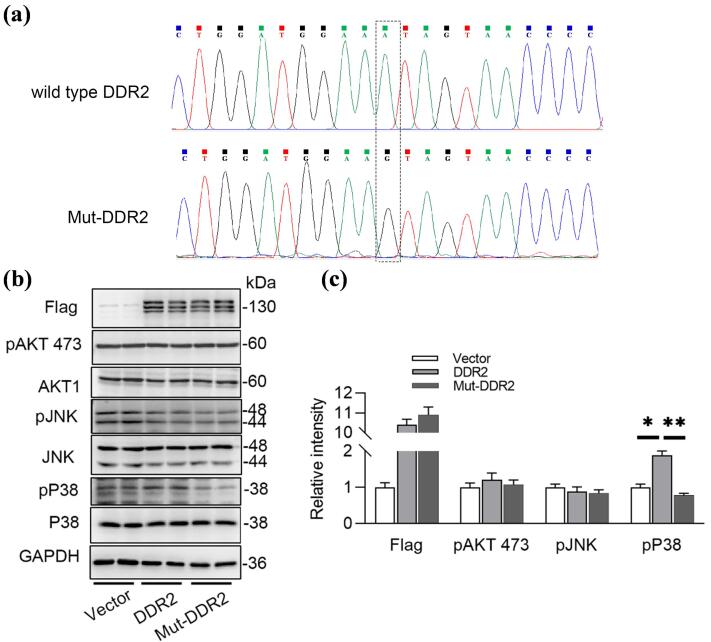
Mutated *DDR2* Failed to Activated the MAPK Pathway.(a) Wild type or mutated *DDR2* (c.431A > G) was constructed into pcDNA3.1 vector; (b, c) Over-expressing wild type *DDR2* or mutated *DDR2* in HEK293wt cells for 48 hours, and examined the AKT and MAPK pathway using Western blot and its statistical graph. ^*^P < 0.05, ^**^ P < 0.01

 Previous studies have reported that *DDR2* is upstream of the MAPK and PI3K/Akt pathways, suggesting that altered expression or loss of function of *DDR2* impacts these pathways. Based on these findings, we conducted plasmids with wild-type *DDR2* or mutant *DDR2* carrying the c.431A > G mutation (Mut-*DDR2*) (Figure 4a), and then transfected them into HEK293WT cells for exploring the differential protein expression. Plasmids with wild type *DDR2* (wt-*DDR2*) (NM_006182) and mutated *DDR2* c.431A > G gene were synthesized by AuGCT Biotech (Beijing). HEK293wt cells were transiently transfected with the recombinant plasmids pcDNA-wt/mutated *DDR2* using Liposomal Transfection Reagent (Lipofectamine 3000, Thermo Fisher Scientific) as per instructions. After transfection, the cells were cultured in fresh DMEM for 48 h, after which total protein was extracted. The proteins were blotted on a nitrocellulose membrane and then incubated with primary antibodies. The membranes were then washed and incubated with a horseradish peroxidase-conjugated anti-mouse antibody or peroxidase-conjugated anti-rabbit antibody (1:1,000; Beyotime, Shanghai, China). The proteins were detected using ECL reagents (Biosharp, Beijing, China). Densitometric evaluation was performed using Image J (NIH, United States). All data are expressed as mean ± SEM, and unpaired t-test was used to assess statistical significance between the two groups. Statistics were computed with GraphPad Prism 8 (GraphPad Software). *P* < 0.05 was considered as statistically significant. As shown in Figures 4b and 4c, only p38 MAPK was activated by *DDR2* overexpression, but not AKT or JNK MAPK pathways. Meanwhile, Mut-*DDR2 *caused a decrease in p38 MAPK activation, indicating a loss of function associated with the mutation.

 We also conducted a literature search on PubMed using the following strategy: ([Discoidin Domain Receptor] OR [Discoidin Domain]) OR [Discoidin Domain Receptor 2] AND [Discoidin Domain Receptor 2 mutation] on July 18, 2024. This search yielded a total of 70 articles. We manually reviewed these articles to identify patients who met the inclusion criteria for WCS. Additionally, we examined the cross-references of relevant articles to ensure comprehensiveness. The final reference list was curated based on relevance to the topic under review. Only articles published in English were included in our review. We identified six previously published cases across three articles between 2006 and 2024. Including the one new case presented in this study, a total of seven cases were available for analysis. Mutations c.1829T > C (p.Leu610Pro) and c.2219A > G (p.Tyr740Cys) in *DDR2 *have been documented.^1,2,4,5^ These mutations affect amino acid residues at intracellular tyrosine binding sites, leading to increased autophosphorylation of *DDR2*, which is hypothesized to underlie the pathophysiology of WCS. Previous cases did not present with clinical symptoms immediately after birth; rather, the symptoms emerged gradually, encompassing skin, joint, ocular, and facial deformities. The genotype and phenotype of all documented patients and our case are summarized in Table 1. Our report describes the first case of WCS with an onset during the neonatal period, highlighting an atypical presentation of this rare syndrome.

**Table 1:** Genotype and Clinical Phenotype of WCS

	**Reference**	**Age**	**Mutation**	**Inheritance**	**Parental affected**	**Eye abnormalities**	**Facial deformities**	**Skin**	**skeletal and joint abnormalities**	**Others**
Our case	after birth		c.431A > G(p.Asn144Ser)	Maternally inherited	No	No	Low nasal bridge, Low nasal bridge, short neck with webbing, low-set ears, short chin. Deformities of posterior nasal choana (left choanal atresia and right posterior stenosis)	Thin skin	laxity in Left hip joint	Small penis, high muscle tension in limbs, 6 × 5 mm solid mass in sacral area, atrial septal defect
Case 1	Warburg et al^1^	57y	c.1829T > C (p.Leu610Pro)	Unknown^a^	No	Small eye fissure, corneal neovascularization, vision loss	Small nostrils, long face, high palatal arch, retroverted ears, micrognathi, aural atresia	Thin skin, keloid scar tissue	Osteolysis Joint contracture	Pneumothorax, Intestinal problems#
Case 2	Cinotti et. al^2^	58y	c.2219A > G (p.Tyr740Cys)	Unknown	No	Small eye fissure, corneal neovascularization, vision decreased	Small nostrils, retroverted ears	Keloid scar tissue, Palmar fibrous bands	Joint contracture, Osteolysis	Conductive hearing loss, mitral valve regurgitation
Case 3	Xu et al^4^	31y	c.2219A > G (p.Tyr740Cys)	*De novo*	No	Corneal neovascularization, Vision decreased	Small nostrils, retroverted ears, long face	Thin skin, keloid scar tissue, follicular keratosis, palmar fibrous bands	Joint contracture, osteolysis, thin ear cartilage, joint swelling	_
Case 4	Xu et al^4^	8y	c.2219A > G (p.Tyr740Cys)	Maternally inherited	Yes	Small eye fissure, corneal neovascularization	Small nostrils, long face	Thin skin, arm papules, palmar fibrous bands	Joint contracture, thin ear cartilage, joint swelling	_
Case 5	Xu et al^4^	3y	c.2219A > G (p.Tyr740Cys)	Maternally inherited	Yes	Corneal neovascularization	Small nostrils, long face	Follicular keratosis	Joint contracture, Joint swelling	_
Case 6	Xu et al^4^	35y	c.1829T > C (p.Leu610P)	Unknown	No	Small eye fissure	Small nostrils, High palatal arch	Thin skin, keloid scar tissue, follicular keratosis, palmar fibrous bands	Joint contracture, osteolysis, joint swelling	Pneumothorax, mitral valve regurgitation, intestinal problems^b^, conductive hearing loss

^a^The parents were not examined by Genomic DNA sequencing. ^b^Intestinal problems include esophageal reflux, pyloric stenosis, chronic diverticulitis.

## Discussion

 Unlike previous cases, which typically manifested during early childhood with normal health at birth and distinctive joint contractures or unusual skin features emerging around 4 to 5 years of age, our case presented symptoms during the neonatal period. Notably, the infant experienced respiratory distress due to upper airway obstruction linked to nasal deformities, along with a short neck and thin skin, suggesting that abnormal bone development already occurred *in utero*. By nine months of age, there was evidence of generalized developmental delay, accompanied with skin lesions. These symptoms are thought to be associated with the mutation in the *DDR2* gene.

*DDR2* is a member of discoidin domain receptors (*DDRs*) family, which constitute a subfamily of receptor-type protein tyrosine kinases (PTKs), and comprises three structural domains: an extracellular domain, a trans-membrane domain, and an intracellular kinase domain (KD).^6^ *DDR2* is predominantly expressed in fibroblasts and mesenchymal cells of various tissues, including the skeletal muscle, smooth muscle, skin, lungs, and kidneys. Its biological functions are largely determined by selective activation through collagen types I, II, III, and X.^7^ The discoidin domain (DS), a homologous sequence in the extracellular binding domain, is a unique structure comprising approximately 155 amino acid residues and functions as a collagen-binding site.^8^ Dimerization of two *DDR2* molecules promotes collagen binding to DS, which triggers phosphorylation of tyrosine residues in the KD.^9^ These phosphorylated tyrosine residues serve as recruitment sites for downstream signaling molecules, thereby activating various signaling pathways, including the MAPK/ERK and PI3K/Akt pathways.^10^ Such signaling cascades regulate critical cellular biological functions, including proliferation, adhesion, migration, and extracellular matrix remodeling.^11^ Genetic mutations in the DS or other regions of *DDR2* may disrupt its signaling pathways, thereby altering cellular behaviors.

 Bone formation is a complex biological process involving intramembranous and endochondral ossification. Runx2, a key transcriptional regulator of osteoblasts, drives the expression of osteogenesis-related genes.^12^ *DDR2* positively regulates osteoblast differentiation through ERK-Runx2 phosphorylation, thus playing a crucial role in osteoblast maturation and homeostasis.^13^ *DDR2* knockout mice exhibit abnormal skeletal development and inhibited chondrocyte proliferation, characterized by dwarfism, short nose, and reduced bone density.^14^ *DDR2 *also regulates chondrocyte proliferation and matrix metabolism during chondrogenesis.^15^ It promotes the transformation of fibroblasts into myofibroblasts with high contractility and extracellular matrix (ECM) secretion capacity, and increases collagen fiber deposition in the ECM by activating downstream signaling pathways (such as p38/MAPK and ERK/MAPK).^16,17^ Meanwhile, abnormally accumulated collagen fibers activate *DDR2*, which induces up-regulation of matrix metalloproteinases (MMPs) and forms a positive feedback loop that accelerates articular cartilage destruction and functional loss.^18^ The clinical features of WCS, such as joint contractures and significant bone resorption in the distal phalanges of the fingers and toes, are thought to be associated with *DDR2* dysfunctions. In our case, the disruption of collagen synthesis or degradation may be associated with defects in nasal structure development, severe limb movement restrictions, and developmental delay at nine months of age.

 Previous cases involved mutations located in the KD, whereas our case features a mutation in the DS. The DS is a crucial component of *DDRs*, harboring collagen-binding sites responsible for collagen recognition and binding, which is a key step in *DDRs* activation.^19^ Upon collagen binding to the DS, conformational changes in the receptor are induced, subsequently activating the KD and initiating downstream signaling, thereby regulating cellular biological functions.^9^ In the present case, the mutation site is located in the DS domain, which is highly conserved in mammals. We hypothesize that this mutation may affect normal collagen-binding, disrupt the proper folding or stability of *DDR2*, and influence protein conformation or interactions, thereby affecting ligand binding or receptor dimerization. Furthermore, we observed that mutated *DDR2* failed to activated p38 MAPK compared to wild type *DDR2 in vitro*, suggesting loss of function of *DDR2* after mutation.

 This case was the first neonatal WCS with a novel mutation in the DS of *DDR2*. The patient presented with significant respiratory distress due to early-life malformations of nasal structures and subsequently developed abnormalities in the skin, bones, and overall development. The absence of certain clinical signs, such as pigmented rashes and corneal neovascularization, may be attributed to the relatively short follow-up duration. This highlights the need for prolonged observation.

## Conclusion

 We report a neonatal case of WCS, thereby expanding the spectrum of clinical phenotype and genotype associated with this disease. Moreover, this case highlights the potential for gene therapy as a future treatment modality.

## Supplementary Files

Supplementary file 1 contains Video S1 (Severe Respiratory Distress of Patient).
